# The complete mitochondrial genome of *Catocala electa* (Vieweg, 1790)

**DOI:** 10.1080/23802359.2020.1772687

**Published:** 2020-06-09

**Authors:** Junxia Zhang

**Affiliations:** School of Biochemistry Engineering, Hohhot vocational college, Hohhot, China

**Keywords:** *Catocala electa*, complete mitogenome, phylogenetic analysis

## Abstract

*Catocala electa* is one of the secondary pests in agriculture. In this study, the whole mitochondrial genome sequence of *C. electa* was determined with the help of Illumina sequencing. The complete genome sequence is 15,575 bp in total length, which contains 13 protein-coding genes (PCGs), 22 transfer RNAs(tRNAs) region, 2 ribosomal RNAs (rRNAs) and 1 control region (CR). The overall base components of mitogenome of all bases was 39.58% for A, 41.72% for T, 11.38% for C and 7.33% for G. Phylogenetic analysis using the ML method showed that *C. electa* was clustered into one branch with Catocala sp. XY-2014

Catocala is a genus of moths in the family Erebidae and is one of the pest groups in agriculture (Fauna Europaea 2011). There more than 250 species distributed in North America in the United States and in Eurasia (Poole 1989) and 150 species distributed in Ancient northern and Oriental realm. In addition, about 100 species were found in the new Nearctic realm. *Catocala electa* is one of the secondary pests in poplars (Tian 2018). In order to identify its taxonomic status and have a highly effective prevention, we collected *C. electa* and sequenced its complete mitochondrial genome.

*Catocala electa* was collected from Hongshan District (42°24′S, 118°91′E) in Chifeng City of China (Inner mongolia, China). The DNA (MBSHISD 20191218) and sample (MBSHIS 20191210) were kept in the Museum of biological specimens of Hohhot vocational college. Samples were deposited at –80 °C for DNA isolation. Total genomic DNA was extracted from the belly tissue using the E.Z.N.A®. Tissue DNA Kit (Omega Bio-tek, D3396-01). Short-insert libraries (insert size 430 bp) were constructed and sequenced on the Illumina Hiseq 4000. The mitochondrial genome was assembled using ABySS v2.0.2 (http://www.bcgsc.ca/platform/bioinfo/software/abyss). PCGs were annotated with homology alignments and *de-novo* prediction. The EvidenceModeler v1.1.1 was used to integrate **the** gene set (Haas et al. 2008). The tRNAs and rRNAs were recognized by the program tRNAscan-SE search server (Lowe and Eddy 1997). The phylogenetic tree construction was carried out based on the Maximum Likelihood (ML) method, using MEGA7.0 (Kumar et al. 2016).

42,295 raw tags with average length of 450 bp and 6,312,011,400 nt were obtained, with total 6,312 Mb data. The complete mitogenome genome sequence is 15,575 bp in total length and contains 13 PCGs, 22 tRNAs, 2 rRNA genes, and 1 control region, showing that the gene composition and arrangement were consistent with Catocala sp. XY-2014 (GenBank: KJ432280.1). The G + C bias accounted for 18.7% in the entire mitogenome. The base composition was as follows: A 39.58%, C 11.38%, G 7.33%, and T 41.72%, respectively. The sequence was deposited in the GenBank (GenBank: MN698265). The phylogenetic position of *C. electa* was investigated to confirm the phylogenetic position, with the concatenated alignments of amino acid in 14 shrimp species using the maximum likelihood method with 1000 bootstrap replicates (Figure 1). *Catocala electa* was clustered with its congeneric species. In this study, the complete mitochondrial genome of *C. electa* was sequenced and annotated to provide important information for Lepidoptera phylogenetic relationships studies.

**Figure 1.**
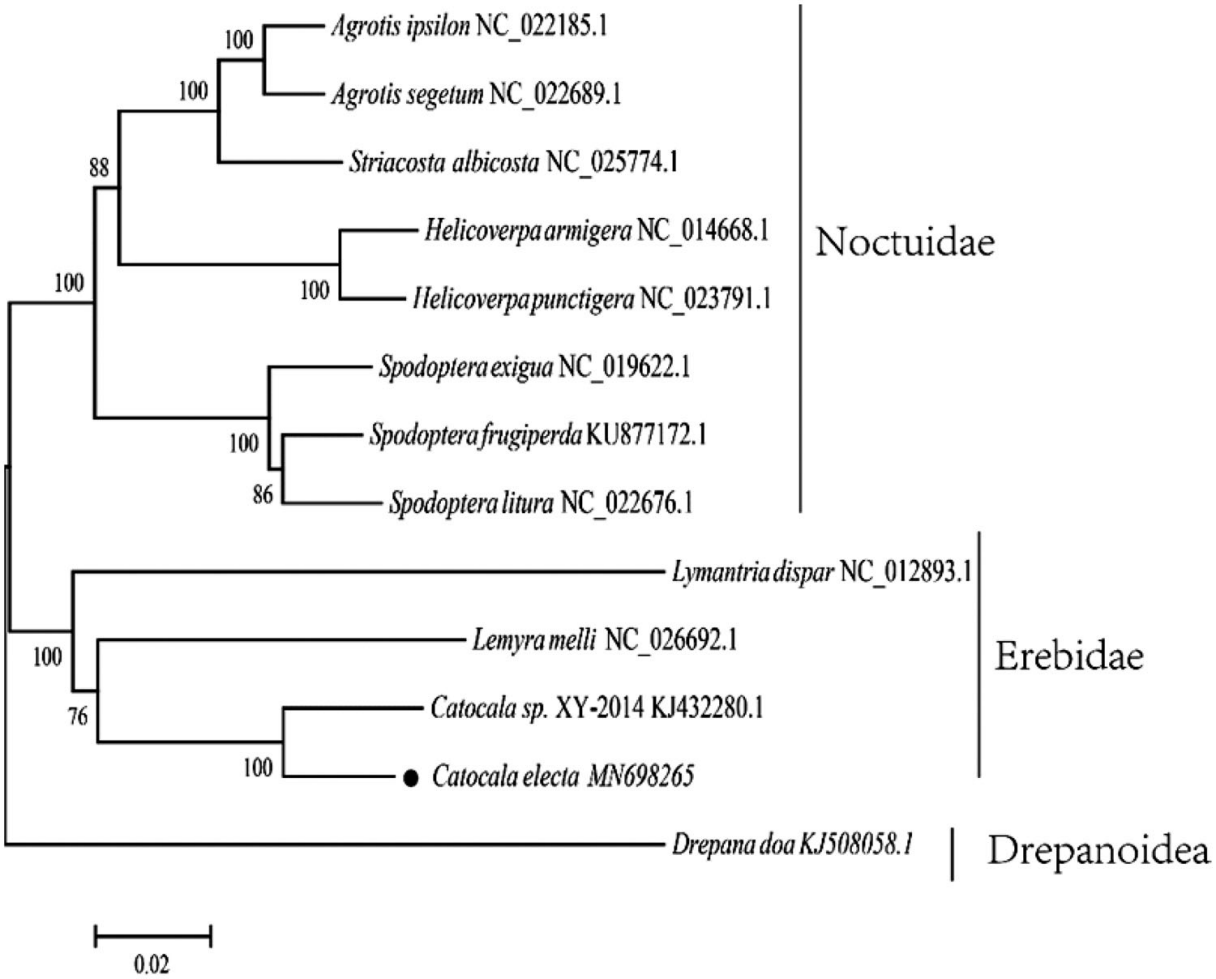
Inferred ML phylogenetic relationship of *C. electa* between other 12 species based on the 13 concatenated mitochondrial PCGs (nucleotide acid data). All the species’ accession numbers in this study are listed as follows: *Agrotis ipsilon* NC_022185.1, *Agrotis ipsilon* NC_022185.1, *Striacosta albicosta* NC_025774.1, *Helicoverpa armigera* NC_014668.1, *Helicoverpa punctigera* NC_023791.1, *Spodoptera exigua* NC_019622.1, *Spodoptera frugiperda* KU877172.1, *Spodoptera litura* NC_022676.1, *Lymantria dispar* NC_012893.1, *Lemyra melli* NC_026692.1, *Catocala* sp. XY-2014 KJ432280.1, *Catocala electa* MN698265, *Drepana doa* KJ508058.1.

## Data Availability

Raw data were generated at on the Illumina Hiseq 4000. Derived data supporting the findings of this study are available on https://www.ncbi.nlm.nih.gov/Traces/study/?acc=PRJNA630527.
